# CT-guided lead placement prevents P-wave oversensing in extravascular ICD

**DOI:** 10.1093/europace/euag134

**Published:** 2026-06-02

**Authors:** Andrea Quaranta, Matteo Baroni, Raffaele Falco, Cristian Martignani, Igor Diemberger, Andrea Angeletti, Carmine Verde, Antonio Nicolò Izzo, Alberto Spadotto, Matteo Ziacchi, Patrizio Mazzone, Mauro Biffi

**Affiliations:** Dipartimento di Scienze Mediche e Chirurgiche, Istituto di Cardiologia, Università di Bologna, Bologna, Italy; Centro Cardiologico De Gasperis, ASST Grande Ospedale Metropolitano Niguarda, Milano, Italy; Centro Cardiologico De Gasperis, ASST Grande Ospedale Metropolitano Niguarda, Milano, Italy; IRCCS Azienda Ospedaliero Universitaria di Bologna, Cardiologia, Via Massarenti 9, Bologna 40138, Italy; Dipartimento di Scienze Mediche e Chirurgiche, Istituto di Cardiologia, Università di Bologna, Bologna, Italy; IRCCS Azienda Ospedaliero Universitaria di Bologna, Cardiologia, Via Massarenti 9, Bologna 40138, Italy; Dipartimento di Scienze Mediche e Chirurgiche, Istituto di Cardiologia, Università di Bologna, Bologna, Italy; Dipartimento di Scienze Mediche e Chirurgiche, Istituto di Cardiologia, Università di Bologna, Bologna, Italy; Dipartimento di Scienze Mediche e Chirurgiche, Istituto di Cardiologia, Università di Bologna, Bologna, Italy; IRCCS Azienda Ospedaliero Universitaria di Bologna, Cardiologia, Via Massarenti 9, Bologna 40138, Italy; Centro Cardiologico De Gasperis, ASST Grande Ospedale Metropolitano Niguarda, Milano, Italy; IRCCS Azienda Ospedaliero Universitaria di Bologna, Cardiologia, Via Massarenti 9, Bologna 40138, Italy

## Introduction

The Extravascular implantable cardioverter-defibrillator (EV-ICD) lead is placed in the anterior mediastinum allowing reliable right ventricular sensing, defibrillation, and pacing while avoiding intravascular hardware.^1^ Nevertheless, results from the Pivotal trial have demonstrated a clinically relevant incidence of inappropriate therapy delivery (IATD), predominantly driven by P-wave oversensing (PWOS).^2^

## Aim

Dedicated algorithms and optimization of lead positioning can mitigate IATD.^3,4^ In our centres, we implemented a CT-guided implantation strategy to position the sensing dipole anteriorly to the right ventricle and maximally distant from the right atrium. This study is based on a prospective observational registry and evaluates the impact of CT-guided lead placement on P-wave detectability, oversensing, and IATD incidence during follow-up.^5,6^

## Methods

### Study design

Prospective, observational, multicenter study conducted at two high-volume EV-ICD implantation centres in Italy. The study complied with the Declaration of Helsinki, and all patients provided written informed consent. Data collection, monitoring, and analysis were performed by the electrophysiology unit staff at each centre.

### Patients and implantation procedure

All consecutive patients undergoing EV-ICD implantation between November 2020 and December 2025 were included (the first 17 patients constituted part of the PIVOTAL study cohort). Planning of the tunnel trajectory was based on a pre-procedural CT scan. The preferred tunnelling pathway was identified to ensure lead placement in front of the anterior RV wall while minimizing the risk of pleural injury and proximity to the right atrial appendage.^6,7^

The defined lead trajectory was marked on the chest wall using CT-derived landmarks, aiming to place the proximal sensing electrode (Ring 2) above the lower RV edge on posteroanterior fluoroscopy and above the cardiac apex in left lateral view. Such placement allowed using the Ring1-Ring2 sensing vector, avoiding extended dipoles (Ring-Can), which are vulnerable to myopotential oversensing. An R-wave amplitude ≥1.0 mV was considered acceptable unless otherwise judged by the operator.

P-wave detectability was defined as an amplitude ≥0.2 mV at intraoperative testing or during follow-up. Oversensing was classified as P-wave, T-wave, or myopotential oversensing based on stored electrograms. The generator was implanted in a subcutaneous or intermuscular pocket, centred or slightly posterior to the mid-axillary line, with its lower border cranial to the ventricular apex. A chest X-ray was obtained before hospital discharge.

### Data collection and follow-up

Baseline clinical characteristics were recorded before implantation. Final lead position was evaluated on post-implantation chest X-rays by measuring the distance between each sensing electrode and a line drawn along the thoracic spinous processes (mid-spinal line). Patients were evaluated during follow-up and with continuous remote monitoring to collect electrical parameters and device interventions.

## Results

### Patient population

**Figure 1:**
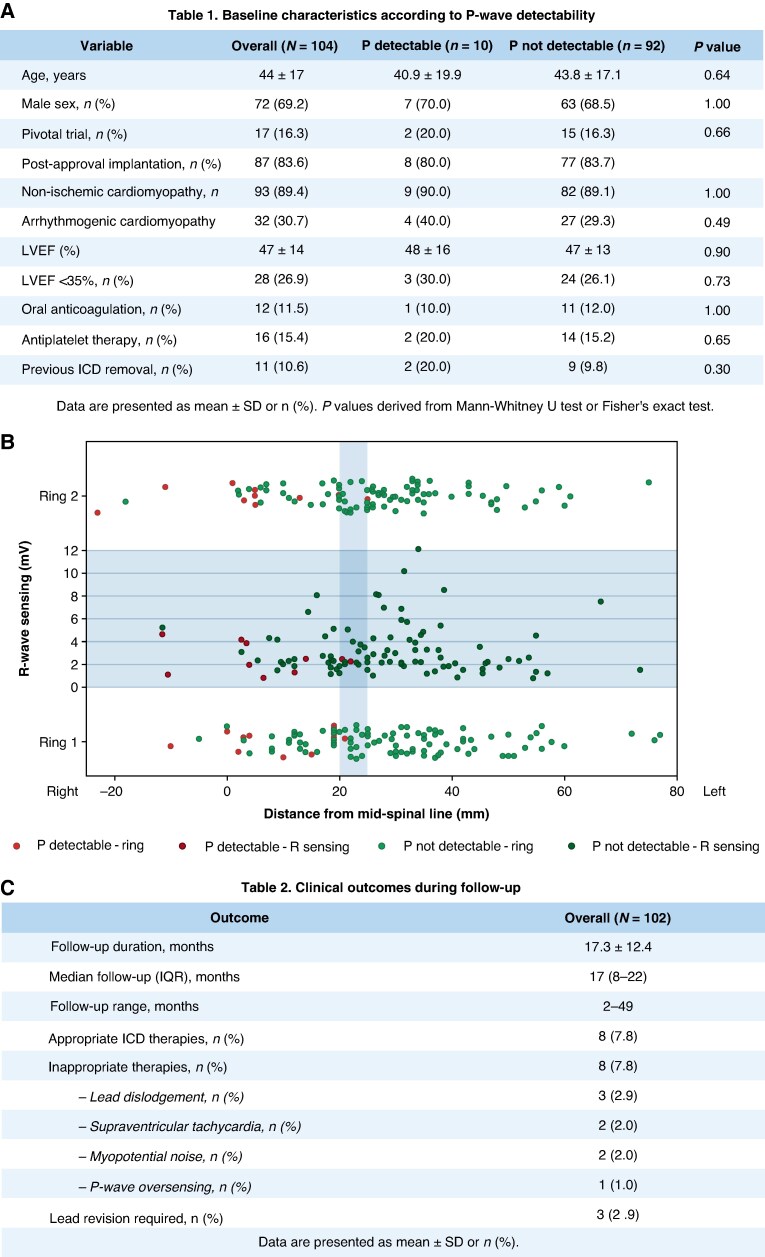
Electrode distance from the mid-spinal line, ventricular sensing, and P-wave detectability. (*A*) *n* = Patient. Baseline characteristics of the study population stratified according to intraoperative P-wave detectability. No clinically relevant differences were observed between patients with detectable and non-detectable atrial signals. (*B*) Scatter plot showing the distance of the two sensing electrodes (Ring 1 and Ring 2) from the mid-spinal line measured on post-implant chest radiography in extravascular ICD recipients. Each patient is represented by three markers aligned along the *x*-axis: the position of Ring 1 (lower row), the position of Ring 2 (upper row), and the corresponding ventricular sensing amplitude (R-wave, mV) displayed within the horizontal light blue band. The R-wave sensing value corresponds to the same sensing dipole defined by the spatial position of Ring 1 and Ring 2. Red dots indicate patients with detectable atrial far-field signals (P-wave ≥0.2 mV), whereas green dots indicate patients without detectable P-waves. Darker tones represent the corresponding R-wave sensing amplitudes associated with the same dipole configuration. A consistent leftward placement of the sensing dipole is observed in patients without detectable P-waves, while ventricular sensing remains preserved across the full range of electrode positions. The vertical light blue band between 20 and 25 mm highlights the transition zone where atrial far-field signals become progressively less detectable. These findings support the anatomical rationale of the CT-guided implantation strategy: positioning the sensing dipole along the left parasternal region, anterior to the right ventricle and maximally distant from the right atrial appendage, reduces atrial far-field signal amplitude while maintaining adequate ventricular sensing. (*C*) Follow-up and device performance.

Baseline clinical and demographic characteristics of the study population are summarized in Table 1 of *Figure 1*.

### Procedural outcomes

102 of 104 patients (98%) were implanted with an EV-ICD: two failed because of inadequate sensing despite multiple tunnelling attempts. The mean procedural time was 93 ± 36 min, mean fluoroscopy time was 5 ± 4 min.

### P-wave detectability and association with electrode position

P-waves were detectable at implantation (≥0.2 mV) in only 10 patients (9.8%), and lead to PWOS with IATD in 1 patient at follow-up.

A strong association was observed between leftward electrode placement and R-wave amplitude/absence of atrial far-field sensing. For Ring 1, the median distance from the mid-spinal line was: 7.0 mm (IQR 2.3–18.0) in patients with detectable P-waves and 29.8 mm (IQR 20.3–38.8) in patients without detectable P-waves (*P* < 0.001, Mann–Whitney *U* test). For Ring 2, the median distance was: 5.0 mm (IQR 1.5–11.0) in the P-wave detectable group and 26.5 mm (IQR 20.0–35.0) in the non-detectable group (*P* < 0.001). The median R-wave amplitude was slightly higher in patients without (2.5 mV, IQR 2.0–4.1) compared with those with detectable P-wave (2.35 mV, IQR 1.3–3.7), albeit non-statistically significant (*P* = 0.35, Mann–Whitney *U* test).

### Follow-up and device performance

Clinical outcomes during follow-up are reported in Table 2 of *Figure 1*. Lead dislodgement occurred in three patients with substernal leads in the early centres’ experience, all obese with sleeve sutured above the rectus abdominis fascia.

## Discussion

Targeting the sensing dipole in front of the RV and distant from the right atrium by CT guidance significantly reduces P-wave detectability and ensures a large R-wave in EV-ICDs (*Figure 1*). A left parasternal trajectory enabled near-complete freedom from clinically relevant PWOS as compared to earlier experiences. Our findings consistently support the anatomical rationale of a CT-guided implantation strategy aimed at maximizing the distance from the right atrium while maintaining lead placement anterior to the RV, which also warrants large R-wave amplitude in the Ring-Ring2 sensing vector. R-wave sensing at implantation showed a broad distribution but was reliably acceptable (generally 2–5 mV), with no apparent reduction in signal amplitude despite increasing leftward placement. These findings suggest that parasternal lead positioning may reduce atrial far-field sensing while preserving adequate ventricular sensing amplitude (*Figure 1*). This latter enhances detection specificity by avoiding the use of Ring-Can dipoles, thereby minimizing myopotential oversensing. Our findings strengthen the importance of integrating pre-procedural imaging with meticulous intraoperative positioning to optimize sensing and minimize inappropriate therapies.

## Conclusion

CT-guided EV-ICD lead implantation enables precise anatomical positioning that minimizes P-wave detectability and myopotential oversensing, thereby reducing the risk of inappropriate shocks. This strategy represents a practical and reproducible refinement of EV-ICD implantation and may improve long-term clinical outcomes.

## Data Availability

Data are available upon reasonable request at the corresponding author.
